# Active Transposition of Insertion Sequences in Prokaryotes: Insights from the Response of *Deinococcus geothermalis* to Oxidative Stress

**DOI:** 10.3390/antiox11030481

**Published:** 2022-02-28

**Authors:** Eunjung Shin, Qianying Ye, Sung-Jae Lee

**Affiliations:** Department of Biology, Kyung Hee University, Seoul 02447, Korea; eunj@khu.ac.kr (E.S.); leavesying@khu.ac.kr (Q.Y.)

**Keywords:** *Deinococcus*, cystine importer, insertion sequence, oxidative stress, redox imbalance, redox signalling, transposition

## Abstract

Bacterial genomes contain numerous insertion sequences (ISs) as transposable elements involved in actions such as the sequestration, transmission, mutation and activation of genes that can influence the responsive capacity of the organism to environmental challenges. To date, at least 30 IS families have been identified. In this review, we describe how certain ISs are transposed to carotenoid biosynthesis genes, such as phytoene synthase and phytoene desaturase, when radiation-resistant *Deinococcus geothermalis* with a redox imbalance and a targeted gene disruption mutation is exposed to oxidative stressors, such as gamma-irradiation, dielectric bilayer discharge plasma and hydrogen peroxide. We also explain the genetic features of IS elements, spontaneous mutation and various stress responses, including nutrient limitation, and physicochemical and oxidative stress, associated with the active transposition of bacterial ISs. Based on the current knowledge, we posit that the redox signalling mechanism inducing IS transposition involves redox sensing and redox switching for the activation of transposase expression and its activity.

## 1. Introduction

Since the discovery of insertion sequences (ISs; also called IS elements) in the late 1960s, the number and diversity of prokaryotic ISs have increased enormously. At present, more than 4600 different ISs in 29 families have been identified and deposited in the ISfinder platform (www-is.biotoul.fr accessed on 15 October 2021) database [1,2]. ISs are integrated as single or multiple copies throughout most bacterial chromosomes, including plasmids, as revealed in genome announcements and recent mobilome studies [3,4,5]. Intragenic transposition, and similarly, intergenic transposition of IS elements can cause gene inactivation. Furthermore, intergenic transposition of IS elements can also affect the expression of neighbouring genes. This is typically mediated by de-repression or by the introduction of a partial or complete promoter located within the IS.

*Deinococcus geothermalis* DSM 11300^T^ is a radiation-resistant bacterium. Its genome contains 73 full-length IS elements from the main chromosome and two mega plasmids. Sixty-eight ISs were extracted to implicate earlier genome states as follows: Forty-two of the IS are integrated into intergenic regions, two in promoter regions and nineteen in open-reading frames (ORFs; e.g., of genes encoding enzymes and ABC transporters), and five in IS elements. Five ISs (e.g., IS*200*/IS*605*) are not extracted in silico because of the single-strand DNA (ssDNA) intermediate transposition without direct repeat sequences (DR) [6]. Disrupted genes could be reassembled, and the order of IS integration across “hot spots” could be determined through in silico correction of mutations caused by the IS element [6]. Subsequent syntenic analysis between *Deinococcus* sp. strain S9 from the microbial mat deposits of hot springs in the Himalaya ranges and *D. geothermalis* from the hot spring at Agnano in Italy revealed over 95% identical amino acid sequences of most genes in the gene arrangement of the longest contig 15 of strain S9. However, different IS elements and additional genes were integrated into the particular region of genomes in both species [6]. Thus, transpositions of IS elements are a major source of genomic plasticity and play a key role in shaping bacterial genomes.

Nowadays, ISs are easily detected and confirmed by comparative analysis of genome sequences using IS determination platforms, such as ISMapper [7], ISQuest [8], OASIS [9], and especially the pioneering database ISfinder [1]. These computational IS-finding platforms provide an IS framework using basic DNA sequence matching in the border regions of IS elements, such as the terminal inverted repeats (TIRs) and the functionally important transposase (Tpase or Tnp) gene, based on amino acid sequence similarity and conserved motifs, like the DDE-motif [2]. As reports accumulated, extracting the information became extremely difficult, so new IS nomenclature and classification had to be established. The ISfinder platform was designed and implemented to maintain a coherent annotation and is now functioning as a reference centre. It assigned nomenclature resembling that used for restriction enzymes: The first letter of the genus is followed by the first two letters of the species and a number (e.g., IS*Bce1* for *Bacillus cereus*; IS*Dge1* for *D. geothermalis*) [2]. According to ISfinder, the *D. geothermalis* genome contains 19 IS types belonging to 9 IS families.

Here, we describe how certain ISs are transposed to carotenoid biosynthesis genes, such as phytoene synthase and phytoene desaturase, when radiation-resistant *D. geothermalis* with redox-imbalance and targeted gene disruption mutations is exposed to oxidative stressors, such as gamma-irradiation, dielectric bilayer discharge plasma and hydrogen peroxide. We also briefly summarise the current knowledge of IS transposition including the genetic features of IS elements, spontaneous mutation and various stress responses, including nutrient limitation, and physicochemical and oxidative stress, associated with the active transposition of bacterial ISs.

## 2. Structural Properties of IS Elements and Functional Aspects

Classical IS elements are between 0.7 and 3.0 kb in length, with one or two ORFs for Tpase and its related protein. Many ISs also contain auxiliary genes that control their transposition. The entire length of the IS is terminated by flanking (im)perfect TIR sequences. Tpase catalyses the DNA cleavages and strand transfers leading to IS transposition [2,10,11]. For example, the IS*1* family classically has two ORFs, InsA and InsB’. InsA negatively controls the expression of the InsAB’-fused ORF for an actual Tpase [12,13]. ISs also often generate short flanking DR sequences at the integration site using the cohesive end of the DNA. Strictly speaking, these DR sequences are not IS components but are key factors for classifying IS elements because some IS elements generate completely conserved DR sequences, some produce variable sequences, and some do not produce DR sequences [2,6]. Although IS classification is based on a variety of characteristics, the amino acid sequence similarity of their Tpases is the principal parameter used for classification [1,14,15,16].

Tpase can be separated into DDE (Asp-Asp-Glu), DEDD (Asp-Glu-Asp-Asp), HUH (His-U-His, U represents a hydrophobic residue), tyrosine (Y), and serine (S) types following the conserved amino acids in the catalytic sites, which affects the chemistry used in breaking and rejoining DNA during transposition. ISs carrying the DDE-type Tpase represent the majority of presently reported IS elements [2]. In *D. geothermalis*, most IS elements are DDE-type, except for IS*Dge10*, IS*Dge18* and IS*Dge19* of the IS*200*/IS*605* family, which use HUH-type Tpase (ISfinder). DDE-type Tpases coordinate essential metal ions and use hydroxyl ions, OH^−^ (e.g., H_2_O), as a nucleophile in a transesterification reaction [2,10]. They do not form covalent Tpase-DNA intermediates during the transposition process. HUH-type Tpases use tyrosine as a nucleophile and generate a transitory covalent 5′ tyrosine-DNA transposition intermediate. HUH-type Tpases are widespread single-strand nucleases [17,18,19] that also include Rep (replication) proteins involved in bacteriophage and plasmid rolling circle replication, as well as relaxases or Mob (mobilisation) proteins involved in conjugative plasmid transfer [20]. Serine Tpase and tyrosine Tpase are relatively minor types.

At present, IS elements in ISfinder are grouped into approximately 30 families, many of which are further divided into subgroups based on shared characteristics. According to Siguier et al., although there are some very well-defined homogeneous families (such as IS*3*, IS*30* and IS*256*; IS*6* and IS*26*) and others that have been redefined over time as ever more IS are identified (e.g., IS*4* and IS*5*), there is no “quantitative” measure of the weight of each of the criteria used to define a family [2,21].

**Figure 1:**
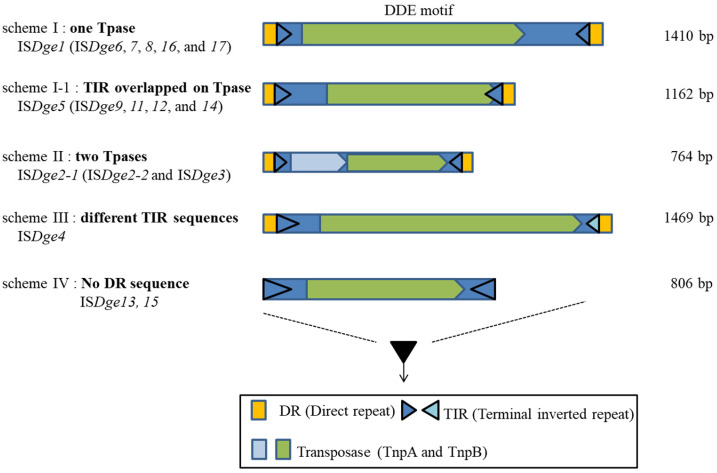
Illustration of four schemes of DDE-type IS elements in *Deinococcus geothermalis*.

The IS*200*/IS*605* family in the HUH superfamily is classified into five structural schemes based on the number of Tpase, Tpase composition and transcriptional direction of Tpase [19]. Thus, we proposed an upper level of IS family clustering and classify four structural schemes of majority DDE motif IS elements following the ORF (mainly Tpase) number, the identity of TIR sequence and DR sequence presence or absence in the case of *D. geothermalis* (Figure 1) [6].

There are two typical IS structures distinguished by the number of ORFs (scheme I–II) and two distinct structures distinguished by the TIR and DR sequence patterns (scheme III–IV). Scheme I ISs have a single Tpase gene and constant DR sequences. Scheme I-1 has a single Tpase gene and an overlapping right TIR sequence in the ORF region of the Tpase. Scheme II ISs have two Tpases-related genes. Scheme III has a typical structural composition, but, interestingly, the TIR sequence is not identical. Scheme IV ISs do not have the two DR sequences in the IS border region.

When this scheme was adopted to DDE-motif IS family classification in *D. geothermalis*, IS*4*, IS*5*, IS*630*, IS*701* and IS*982* belonged to scheme I, the IS*1* family belonged to scheme II, IS*66* belonged to scheme III and the IS*6* family belonged to scheme IV. The IS*6*/IS*26* family has a rule in their TIR sequence, such as “GG-4N-G”, which means that the first two nucleotides are “GG” and the seventh nucleotide is also “G”. The IS*6*/IS*26* family included IS*Dge13* and IS*Dge15* of *D. geothermalis* [22]. It was proposed that all the collected IS*6* and IS*26* families of bacterial and archaeal genomes encompassed a single family. However, IS*Dge13* has a duplicated “GG-4N-G” conserved sequence in the TIR sequence because of target site duplication: Either the DR sequence is not detected, or the DR sequence is eight nucleotides in length (ISfinder). Interestingly, there were different DR sequence absence ratios of IS*6* family members in 88% of Gram-negative bacteria, 60.6% of Gram-positive bacteria and 35.1% of archaea, according to Harmer and Hall’s data [22]. Recently, Varani et al. also reviewed the IS*6* family, specifically, the Tpase size compositions and the structures of 160 IS*6* family members important in generating multiple antibiotic-resistance genes in bacteria and archaea [23]. Additional transposition experiments are needed to detect the transposition events in IS*6*/IS*26* family members for further classification of these IS elements within prokaryotic genomes. The next section looks briefly at the transposition mechanisms of IS elements.

## 3. IS Transpositional Procedures and Trigger Factors

In general, IS transposition processing is dependent on the type of Tpase in the IS family. Most DDE-motif Tpases have a “copy-and-paste” mechanism [2,10,11]. In the case of *D. geothermalis*, the active transpositions of several IS elements of the IS*1*, IS*5*, IS*6* and IS*701* families perform this “copy-and-paste” or a replicative mode of transposition [24,25,26]. Briefly, the expressed Tpase bound to itself on the end of the IS element and cleavage covalent bond between adjacent nucleotides and the template DNA synthesised nascent strand in both strands. The excised double-stranded DNA-Tpase complexes bind to the target site and integrate in a manner similar to the sticky ends produced by restriction enzymes [2]. The sticky ends for novel integration became DR sequences in the border region of IS elements. There are several other modes of active transposition besides the “copy-and-paste”. For example, the IS*4* family of IS*10*/IS*50* shows the “cut-and-paste” mode, and IS*91*/Tn*916* shows no DR sequence production and performed a rolling-circle mechanism [2].

For IS*200*/IS*605* family members, the transposition mechanism is well understood due to a combination of genetic, biochemical and structural studies [27]. Briefly, it can be described as a single-strand “peel-and-paste” mechanism in which the IS is excised as a single strand (the “top” strand) from the lagging strand template of the donor molecule to form a single-strand transposon circle and then is inserted into a single-strand target at the replication fork [19]. The IS*200*/IS*605* family members include subterminal secondary structures recognised by TnpA. The cleavage sites occur a short distance of 5′ to the left and 3′ to the right of the structure. These are not directly recognised by TnpA but form a complex set of interactions with the internal sequence that permits their cleavage. Transposition occurs by insertion of the left end 3′ to a specific tetra- or pentanucleotide essential for excision and further transposition. Insertion does not generate a DR and occurs preferentially in the lagging strand template of the replication fork. For example, IS*608* from *Helicobacter pylori* always inserts 3′ into a TTAC tetranucleotide, and IS*Dra2* specifically integrates 3′ into a TTGAT pentanucleotide. This results in a clear orientation bias at the genome level, reflecting the direction of replication of the target replicon. The success of these IS elements is related to the interplay between Y1 HUH Tpase (an ssDNA endonuclease that contains only one tyrosine active site) and replication fork-associated factors and can be detected in numerous bacterial genomes. [18,19,20,27,28]. Moreover, IS*Dra2* transposition is intimately associated with the gamma-irradiation-induced genome fragmentation and reassembly of *D. radiodurans*. In this section, we provide a summary of IS transposition studies in various species of prokaryotes and some interesting perspectives.

### 3.1. IS Transposition in Deinococcus-Thermus

Although the genome of the hyperthermophilic bacterium *Thermus scotoductus* contains the highest IS copies in terms of the genome size, the genomes of 3 of the 28 *Thermus* spp. (*Thermus thermophilus* HB8, HB27 and NAR1) contain a broad spectrum of eight IS family members: IS*3*, IS*4*, IS*5*, IS*1634*, IS*256*, IS*630*, IS*701* and IS*110* [3]. Interestingly, IS*Tth3* of the IS*1634* family, IS*Tth4* of the IS*256* family, IS*Tth7* of the IS*5* family and IS*1000A* of the IS*110* family transpose at a relatively high frequency in *Thermus* spp. The functional roles of IS*Tth7*, which is actively transposed by streptomycin, were identified by Gregory and Dahlberg [29]. IS*Tth7* inserted in the *rsmG* gene encoding a methyltransferase that produces m^7^G527 in the 16S rRNA, resulting in the streptomycin resistance phenotype. IS*Tth7* is widely distributed in the genomes of *T. thermophilus* strains in the form of a potentially activated full-length IS element [3].

When *D. radiodurans* was exposed to gamma-irradiation, IS*Dra2* of the IS*200*/IS*605* family was inserted into the *thyA* gene encoding a thymidylate synthase, which was selected under appropriate conditions on a trimethoprim (100 µg/mL) plate [17,18,19,27,30].

**Figure 2:**
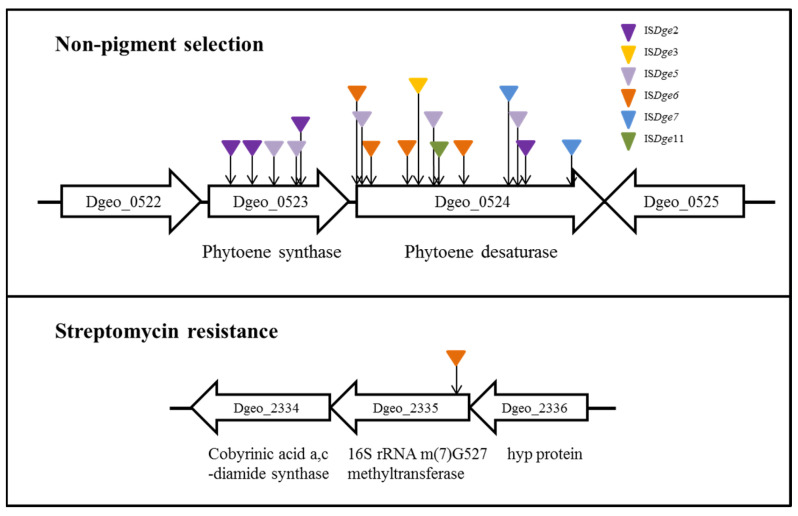
Active transposition of IS elements in *Deinococcus geothermalis* under oxidative stress. The transposition events were isolated from two phenotypic selections.

Among different *D. geothermalis* lineages, the following specific types of IS elements actively transposed to carotenoid biosynthesis genes (phytoene cyclase and phytoene desaturase) and an *rsmG* gene (conferring streptomycin resistance) with a replicative mode due to hydrogen peroxide-induced oxidative stress conditions: IS*Dge6* of the IS*5* family in a LysR family regulator-disrupted mutant, IS*Dge5* of the IS*701* family in a *dps* gene (encoding a chromosome stabilizer protein, Dps: DNA-binding protein from starved cells)-disrupted mutant strain and a cystine importer-disrupted mutants and IS*Dge11* of the IS*4* family in the wild-type strain (Figure 2) [24,25,26]. Nevertheless, the scheme III transposition events of IS elements with different TIR sequences and the scheme IV transposition events of IS elements without DR sequences have not yet been found under the oxidative stress condition in WT and target gene-disrupted mutants [31]. If the transposition of particular IS elements is under the influence of unique target genes, it will be possible to explain and understand the states of IS induction for sensing and signalling environmental stress and genome evolutionary processes in prokaryotic genomes.

### 3.2. IS Transposition in Gram-Negative Bacteria

Here, we focused on the well-defined transposition cycle of IS*911*. IS*911* of the IS*3* family was transposed to the phage *cI* repressor gene in *Shigella dysenteriae* phage λ lysogen by spontaneous insertion [32]. This IS*911* element is found in the genome of various Gram-negative bacteria, including *Escherichia coli* K12. Scheme II IS*911*, containing DDE motif Tpase has an overlapping right TIR sequence in the Tpase ORF, may need to extend scheme II-1. Temperature-induced (42 °C) transposition of IS*911* formed figure-eight molecules [33]. This transposition procedure is the “copy-out-paste-in” mechanism. Interestingly, IS*911* expressed the fusion protein OrfAB by frameshifting through “slippery” lysine codons, and Tpase expression was controlled by the “junction” promoter that assembled in both TIRs following the circulation of IS*911*. The overall two-step IS*911* transposition required the consecutive assembly of synaptic complex A (SCA) to start the process, leading to replication and circularisation of the transposing IS copy, and synaptic complex B (SCB) to ensure that the replicated copy was integrated into the target DNA. In the first step, OrfAB Tpase is bound to the TIRs, then assembled by end pairing, followed by one-end cleavage and a strand transfer to form figure-eight molecules for excision of the IS element. In the second step, the circular IS*911* was generated by replication and target pairing with the involvement of the OrfAB complex through one-end cleavage and strand transfer, then inserted in the target loci. IS*911* insertion is also possible at nontarget loci due to the collaboration between OrfAB and OrfA via the two-end cleavage and strand transfer [32,33].

Transposition procedures of ISs occurred in different ways dependent on the IS family in numerous Gram-negative bacteria, and the triggering factors of transposition are also varied. For example, *Pseudomonas* and *Burkholderia* respond to high temperatures, conjugation or oxidative stress resulting in active transposition of IS elements [34,35,36].

### 3.3. IS Transposition in Gram-Positive Bacteria

Transposition of various ISs, such as IS*4*, IS*701*, IS*1634* and IS*Lre2*, has been described in *Geobacillus kaustophilus* via *sigX*-dependent stress responses at elevated temperatures, under nutrient limitation for uracil prototrophs, C- and N-source starvation and antibiotics treatment [37]. A mobilome analysis of 102 genomes of *Bacillus cereus sensu lato* species revealed 16 IS families distributed in a species-dependent manner [4]. For example, IS*982*, IS*630* and IS*5* were uniquely located in *Bacillus* *thuringiensis*, whereas IS*4* and IS*3* were distributed among all analysed genomes. Moreover, *Bacillus* *subtilis* 168, which has no IS*4Bsu1*, exhibited IS*4Bsu1* transpositions from *B. subtilis* (*natto*) when grown under a high temperature and competence-inducing conditions but not under an optimal temperature and nutrient-rich medium conditions [38].

### 3.4. IS Transposition in Archaea

Filée et al. reviewed archaeal IS diversity. Archaeal genomes contain numerous IS families, just like bacterial genomes [39]. Although genome sequence analysis has detected many IS elements, there are rare observations of IS transposition via an induction mechanism, except for spontaneous mutation [40]. Perhaps the archaeal active transposition of IS is a prominent case study in this evolutional research field [41].

### 3.5. Use of Transposon Mutagenesis

Typically, transposon (Tn) mutagenesis is a powerful tool for detecting the functional role of uncharacterised genes using random transposition selection by Tn. In *Bifidobacterium longum*, IS*Blo11* of the IS*3* family was detected by the *sacB*-based counterselection system and analysed for its activity in *E. coli*. The constructed *E. coli*−*Bifidobacterium* shuttle vector harbouring *sacB* was introduced into *B. longum*. IS*Blo11* moved into *sacB*, and the sucrose-resistant phenotype was selected. IS-transposed clones in *B. longum* 105-A were selected by simple conjugation in *E. coli* and growth in 4% xylose and 1% glucose [42,43].

## 4. Inductive Signals of Active Transposition

In many IS elements, a single Tpase controls its own IS expression and transposition by its own promoter. However, in the case of IS elements consisting of two ORFs, for example, *tnpA* encodes a Tpase and *tnpB* encodes a repressor resulting in IS*Dra2*, transposition is regulated by inhibiting excision and insertion of whole IS elements [18,19,27]. The expression level, protein stability and activity of a particular Tpase are important factors for the successful transposition of a particular IS. Together with different host factors, these Tpase-associated factors are controlled by transcriptional and translational regulation [44].

However, the question remains: What are the Tpase induction signalling pathways that sense environmental stressors? Some of the many possibilities are host factors, such as directly bound chromosomal DNA stabilisers; redox-sensing regulators, including reactive oxygen species (ROS)-sensing; particular signal transduction pathways; intracellular low-molecular-weight (LMW) compounds, such as thiols and redoxins; conjugation; cellular toxic physical and chemical factors, as well as spontaneous transposition [16]. Here, we summarised several factors, including host factors, environmental factors, such as temperature and nutrition, ROS-producing radiations, redox imbalance and some known redox-signalling events by oxidative stressors.

### 4.1. Host Factors

Nucleoid-associated proteins that induce changes in DNA structural topology can affect the behaviour of IS elements. For example, H-NS (histone-like nucleoid structuring protein) may explain the targeting preferences of H-NS mutants for IS*903* and IS*10* [45], IS*1* [46] and IS*5* [47]. H-NS is necessary for efficient IS transposition [47,48]. In addition, IS transposition can also be modulated by host DNA stabilisers, such as IHF (integration host factor), HU (heat-unstable protein) and FIS (factor for inversion stimulation), the replication initiator DnaA, the protein chaperone/protease ClpX/P/A, the SOS control protein LexA, the DNA methylase Dam and GTP levels [44,48,49,50]. Nevertheless, the in vitro detection method of some host factors of active transposition is limited because the screenings always have a positive effect. General host factors have affected chromosome stabilising, resulting in the need for multiple-level signalling for IS transposition.

Bacterial IS transposition reactions have also been shown to be regulated by Hfq. This well-studied RNA chaperone binds small regulatory RNA (sRNA) and plays a central role in complex post-transcriptional regulatory networks for target genes in many bacteria [51,52,53]. For example, it interacts directly with the ribosome-binding site of IS*10* Tpase mRNA, resulting in translation inhibition [54].

Dps works in concert with other host factors to organise the chromosome. It is abundant in starved *E. coli* cells and is involved in stress resistance. Dps is dominantly expressed in the stationary phase of *E. coli* and affected IS transposition, although the exact mechanism is not yet known. In the case of *D. geothermalis*, *dps*-deficient mutants exhibited active transposition of specific IS family members [24].

The transpositional activity of IS elements is under strict regulation, presumably to limit their effects on the host cell. Vandecraen et al. aptly summarised the regulatory factors controlling IS transposition [16]: Transcriptional repressors [55,56], translational inhibitors [57], ribosome frameshifting [58], impinging of transcription by secondary mRNA structures [59], methylation sites [60], Tpase instability [61] and target site preference [62,63].

### 4.2. Nutrition and Temperature

The nutrient-rich environment (e.g., high-glucose level) of the host reduces the requirement for many genes that are essential for free-living bacteria. This allows the fixation of slightly deleterious mutations in the population by random stochastic IS transposition and the concomitant increase in the IS copy number in the genome [2,64].

In *E. coli*, the IS*1*, IS*30* and IS*911* families show temperature-sensitive transposition [46,65]. Specifically, high-temperature conditions decreased the transposition activity. In contrast to *E. coli*, the transposition of novel IS elements of IS*5* and IS*21* family members from a soil-derived *Burkholderia multivorans* strain was enhanced 7-fold under a high temperature at 42 °C but not under oxidative stress and starvation conditions [34]. Genome-wide sequencing and Southern blot analysis of IS elements revealed that the *G. kaustophilus* wild-type genome contained 19 IS families and 118 copies of full-length IS elements. Several IS families showed growth inhibition-, sigma factor- and heat shock-dependent active transposition [37].

Using the papillation screen method in *E. coli*, various stressors positively affected IS transposition by different host factors, such as regulators, metabolism, protein stability and folding and DNA metabolism [51]. In conclusion, the IS transposition induction in prokaryotes is a complex and orchestrated phenomenon that is affected by a large number of host factors and environmental factors.

### 4.3. Gamma-Irradiation and Dielectric Bilayer Discharge Plasma

Gamma-irradiation is a strong DNA-damaging agent that results in genome DNA fragmentation. Interestingly, in this extreme stress state of *D. radiodurans*, five IS elements (IS*2621*, IS*Dra2*, IS*Dra3*, IS*Dra4* and IS*Dra5*) are actively transposed to the *thyA* gene by gamma-irradiation [19,30,66]. Under ultraviolet radiation, the transposition of IS*2621* into the *uvrA* gene encoding for replication, repair and recombination factors was detected [67]. IS*Dra2* of the IS*200*/IS*605* family is induced 100-fold by gamma rays [66].

We recently performed gamma-irradiation treatment of *D. geothermalis* WT and several targeted gene-disrupted mutants. Our carotenoid-deficient screening test successfully obtained non-pigment mutants with the disruption of the two genes encoding phytoene cyclase and phytoene desaturase, respectively, caused by the IS integration. Gamma-irradiation led to additional transpositions of IS families compared to the oxidative stress response to H_2_O_2_. However, each IS family still followed a specific selectivity (Ye et al., submitted). For example, IS*Dge2* and IS*Dge3* of the IS*1* family are limitedly transposed by gamma-irradiation. In addition, the dielectric bilayer discharge (DBD) plasma-radiation was also performed to detect active IS transposition. This approach successfully increases the frequency of active IS transposition to the target genes. Interestingly, these IS transposition events of DBD plasma-radiation are the same IS types and transposition loci as those under H_2_O_2_-induced oxidative stress.

### 4.4. Redox Imbalance

**Figure 3:**
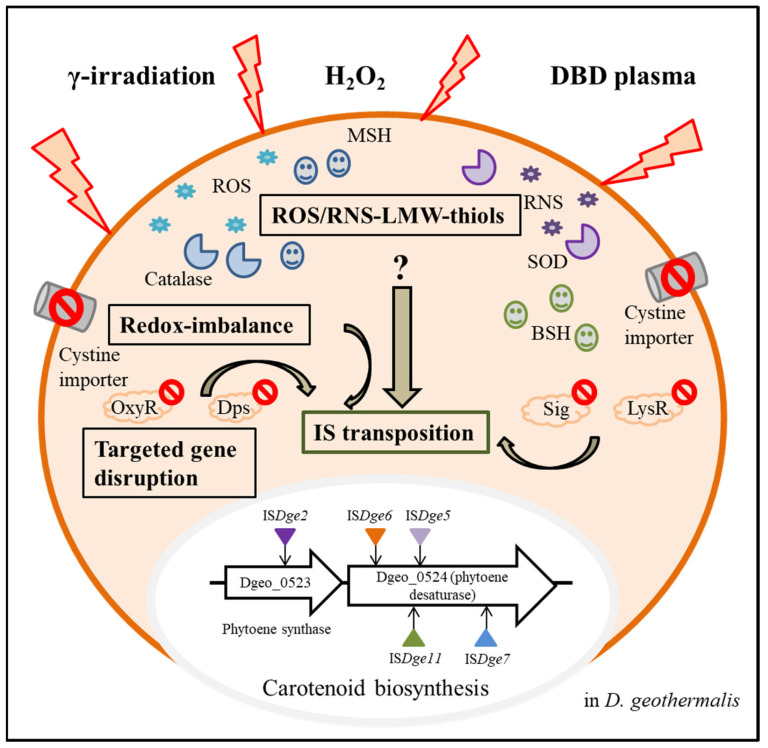
Overview of active transposition of ISs through various intracellular factors in *Deinococcus geothermalis* from three extracellular stressors. Three extracellular stressors induced ROS and RNS, and a cystine importer-disrupted mutant induced the intracellular redox imbalance state. LMW-thiols, such as bacillithiol and mycothiol, and antioxidant enzymes were produced. As a result of oxidation stress in WT and mutants with targeted gene disruption of *oxyR*, *lysR*, *dps* and *sig*, particular IS elements were transposed to the carotenoid biosynthesis genes, and phenotypic non-pigment colonies were isolated.

Furthermore, we additionally demonstrated specialised IS transposition in the redox-imbalance condition using a cystine importer-disrupted mutant and its complementary strain [26]. Interestingly, both IS*Dge5* of the IS*701* family and IS*Dge7* of the IS*5* family transposed to carotenoid biosynthesis genes, resulting in the non-pigmented phenotype (Figure 3). This selectable IS transposition mirrors the phenomenon of the *dgeo*_0257 (DgDps3)-disrupted mutant [24]. In the redox-imbalanced state, the expression level of DgDps3 was enhanced, but DgDps1 was strictly downregulated. DgDps1 and DgDps3 showed complementary effects on their expression levels depending on the growth phases. DgDps1 expression was enhanced during growth in the absence of DgDps3. Similarly, DgDps3 was strongly expressed at the early and late exponential growth phases in the DgDps1-deficient condition under oxidative stress (Bae et al., manuscript prepared). DgDps3-deficient conditions revealed active transposition of IS*Dge5* of the IS*701* family. Both redox-imbalance and *dps*-deficient conditions often revealed a common transposition phenomenon of the IS element. This leaves a pertinent question regarding Tpase induction signalling and its network regulation between redox-imbalance and *dps*-deficient conditions under oxidative stress. Interestingly, *dps* gene-deficient mutants no longer expressed *bshA*, a gene required for bacillithiol biosynthesis [31]. However, WT and redox-imbalanced strains revealed similar expression levels of *bshA* [26]. Under oxidative stress, *dps*-deficient mutants revealed dramatically enhanced expression of mycothiol biosynthesis enzymes, such as MshBCD, specifically at the early exponential growth phase. Therefore, specific redox-sensing signal transduction for LMW-thiols-mediated induction could modulate the redox-sensing regulators involved in the oxidative stress response. However, deconvoluting the signalling pathways from ROS sensing to activating IS transposition remains a challenge.

### 4.5. Antibiotics

Recently, we applied oxidative stress-induced IS transposition to streptomycin-resistant phenotypic selection because antibiotic resistance is a powerful selectable biomarker, and the genetic network of the target genes and biochemical functions are normally well defined. Generally, peptide translocation-blocking aminoglycoside streptomycin was affected on the ribosomal structure via direct interaction with its component’s ribosomal proteins and 16S rRNA [68]. The streptomycin-resistant phenotype is easily obtained through low-level streptomycin induction, resulting in spontaneous mutations in ribosomal component-related genes, such as *rpsL* encoding a S12 ribosomal protein, *rsmG* which is a chemical modifier of m^7^G527 on the 16S rRNA and *mthA* encoding an enzyme involved in the S-adenosylmethionine (SAM) recycling pathway (see Section 3.1) [69,70]. These spontaneous mutations revealed MIC (minimum inhibitory concentration) level-dependent resistance [71]. Interestingly, when we induced oxidative stress by H_2_O_2_ treatment in a LysR family regulator-deficient mutant, the IS*5* family element was actively transposed to *rsmG*, resulting in strains with high streptomycin resistance (MIC > 10,000 µg/mL; Lee et al., submitted).

This observation is similar to particular IS transposition in the thermophilic bacterium *T*. *thermophilus*. Researchers performed serial cultivation with gradual streptomycin treatment and selected the streptomycin-resistant phenotype, which originated from many spontaneous mutations and the transposition of the IS*Tth7* to *rsmG* [29,72]. Surprisingly, transposition of the IS by oxidative stress (H_2_O_2_ treatment) for streptomycin resistance was limited in a LysR family regulator-deficient mutant.

### 4.6. Metals

The genome of the famous metal-resistant bacterium *Cupriavidus metallidurans* CH34 contains 21 distinct IS elements (a total of 57 full-length ISs) belonging to 10 IS families [73]. When zinc ions (Zn^2+^) at an 0.8 mM concentration were present in the culture plates, zinc-resistant mutants from *C. metallidurans* AE126 (a strain cured for pMOL30 mega-plasmid) with a compromised *czc* (cadmium/zinc/cobalt resistance) operon were isolated. Vandercraen et al. performed colony PCR experiments to determine the active transposition of IS elements affecting the *cnr* (cobalt-nickel resistance) operon and found the transposition of mainly IS*Rme5* and another six IS elements in the first population [74]. Moreover, the second population enhanced the contribution of IS elements with the identification of IS*1087B* and IS*1088*, in addition to IS*Rme5*. Interestingly, these major three IS elements increased the endogenous promoter activity of Tpase via Zn^2+^ and Cd^2+^ (cadmium ion) induction. Further research is needed to understand the relationship between metal ion stress and the transposition of IS elements.

## 5. Redox-Switched Regulators and Redox Signalling

Currently, there is no clear evidence that the multiple stress-sensing regulators directly cause IS induction. Is this because of what happens in various stressed cells, including oxidative stress, and which triggering factors selectively activate particular types of IS element silencing in the genomes?

Very recently, chromatin immunoprecipitation sequencing (ChIP-Seq) analysis and RNA sequencing (RNA-Seq) analysis showed that the radiation/desiccation response (RDR) regulon operated through the *cis*-acting sequence RDRM (Radiation Desiccation Response Motif), the *trans*-acting repressor DdrO and the protease IrrE possibly controlled a unique IS family [75]. The authors performed a systematic and comprehensive sequence analysis for the RDR motif and newly found the DdrO target genes, which involved two Tpase that included the IS*5* and Tn*3* families. The DdrO-IrrE regulatory system is a recently well-defined regulatory system in the extremely radiation-resistant bacterium *D. radiodurans* [76,77,78]. When cells were exposed to oxidative stress, most of the ROS produced was directly detoxified by the enzymatic cooperation of catalase, peroxidase and superoxide dismutase and the scavenging of LMW thiols. Nevertheless, ROS triggered the chemical modification of several transcriptional regulators, resulting in the activation of proteins, such as the redox-sensitive transcriptional regulator OxyR. When cells were exposed to radiation and desiccation stresses, the intracellular redox balance was destroyed and the Zn^2+^-chelating proteins were distorted, releasing Zn^2+^. Zinc is a cofactor element for the IrrE protein. IrrE is a metalloprotease that cleaves the dimeric form of DdrO bound to the RDRM motif of the RDR regulon. By inactivating the repressor DdrO, gene expression mediated by the RDR regulon is activated [75,78]. We expect this is an example of a defined redox-sensing pathway. Perhaps multiple signalling pathways for sensing a redox imbalance are connected to different environmental stressors. Logically, many transcriptional regulators recognise redox changes in the cytoplasm, and particular regulators might control the expression of Tpase as a key player in IS transposition [79,80,81]. Recently, the proteome data of *Deinococcus* were updated, thus enabling the use of this model organism for investigating multi-scale proteomic questions, including how response mechanisms cope with physicochemical stresses [82,83]. This information provides research opportunities to identify the redox-signalling pathways and regulators of active IS transposition.

## 6. The Evolution of Prokaryotic Genomes via IS Elements

There are many hypothetical backgrounds for bacterial genomic evolution. The proposed extracellular origin is based on horizontal gene transfer mechanisms. Prokaryotic cells acquire foreign DNA through several gene transfer systems. The lateral gene transfer of genes can occur through transformation, transduction and conjugation. The intracellular mechanisms for genome plasticity are gene duplication by recombination, deletion, homopolymeric tracts by the slippage of DNA polymerase [84] and the moving of transposable elements. The impact of IS transposition on the bacterial genomes can have positive, neutral or negative effects on cell fitness. Under selective conditions (e.g., antibiotic pressure), IS-mediated beneficial mutations are favoured, and fixation of the IS at that particular target site depends on its exerted effect (e.g., gene inactivation or modulation of gene expression).

Perhaps the most common effect of IS transposition is gene inactivation. Many cases have been described illustrating the modulation of antibiotic and xenobiotic resistance, virulence and metabolic activity modulation by IS-mediated gene inactivation. IS transposition to non-coding regions can lead to the altered expression of neighbouring genes by IS elements carrying a complete outward-directed promoter [16].

Vandecraen et al. [16] posited four hypotheses as an interesting question regarding how ISs are maintained in a bacterial genome on an evolutionary timescale: The parasitic nature with self-replicating ability, generating occasional beneficial mutations in promoting genetic variation, selectively neutral and coexistence in a dynamic equilibrium.

IS elements can have an important impact on the evolution of their hosts. In the case of *D. geothermalis*, chronic H_2_O_2_-exposed cells exhibited the streptomycin resistance phenotype due to IS transposition and several point mutations in streptomycin resistance-related genes, such as *rpsL* and *rsmG* (Lee et al., submitted). In particular, streptomycin-dependent phenotypic strains grew better in the presence of streptomycin and under the oxidative stress condition compared to the wild-type strain. Thus, whether or not IS elements are selfish and parasitic, their impact on the architecture of microbial genomes regarding adaptation and survival is undeniable.

The insertion of specific IS elements, such as IS*1*, IS*3* and IS*5*, can result in topological changes in the stress-induced DNA destabilisation (SIDD) region near the promoter as a “hotspot” and transcriptional activation of the *flhDC*-encoded master regulator in the flagella system [85,86]. In *E. coli*, the normally silent β-glucoside (*bgl*) catabolic operon, the *glpFK* for the glycerol utilisation operon and a cryptic anabolic functional operon are activated by the insertion of IS*5* upstream of the promoter under starvation in the presence of glycerol, and specific genes encoding nitroreductases are inactivated under antibiotic stress [16,47]. IS*5* can lead to gene activation via integration into a specific recognition sequence in the SIDD region, and precise excision of IS*5* from the *fucPIK* operon can reverse the activation [87]. Humayun et al. reported that environmental stress conditions appeared to influence IS*5* insertion through the relatively non-specific nucleoid protein H-NS and locus-specific DNA-binding proteins, such as GlpR and Crp [47]. These findings provide supporting evidence for a highly evolved and mutually beneficial relationship between IS and the host genome.

We expect that studies of the active transposition of ISs in *Deinococcus* as a model system will explain the molecular evolution of genome plasticity in the imminent future. In particular, the classification of active transposition of IS types in WT and targeted gene disruption mutants offers a clue to define specific IS selectivity and functional regulatory networks between intracellular redox-imbalance and redox-sensitive regulators (e.g., a LysR family member, a sigma factor and a putative Dps protein) under differential oxidative stress-inducing treatments.

## 7. Conclusions

In this review, we covered the active transposition of IS elements in prokaryotic genomes. The activation of IS transposition was triggered by various extracellular and intracellular redox imbalances, including oxidative stress and physicochemical stress. Currently, there is a gap linking the redox-sensing regulations with the active IS transposition of prokaryotes. In the near future, it is expected that further mobilome analysis and full genome sequence analysis by deep sequencing and IS-sequence technology will assist in understanding the IS distribution and stressor-dependent IS transposition patterns in several natural isolates of the same bacterial species. Therefore, *D. geothermalis* is a good model organism for explaining the network regulations, including the active transposition of IS elements, redox-sensitive regulators and intracellular stress responses.

## Abbreviation

IS	insertion sequence
Tpase (Tnp)	transposase
InsA; InsB’	two ORFs in IS element and in some cases both genes were produced a fused protein (InsAB’) or InsA regulated InsB’ expression
TIR	terminal inverted repeat
DR	direct repeat
Dps	DNA-protection protein from starved cell
H-NS	histone-like nucleoid structuring protein
LysR	a broad transcriptional regulator family
DBD	dielectric bilayer discharge
DdrO-IrrE	*Deinococcus* unique regulatory system for RDR regulon
RDR	radiation-desiccation responded regulon
ROS	reactive oxygen species
SIDD	stress-induced DNA destabilisation region
